# Risk perception of patients with chronic illnesses toward the SARS-CoV-2 in northeastern Ethiopia in 2020

**DOI:** 10.1016/j.pmedr.2024.102751

**Published:** 2024-05-09

**Authors:** Dejen Getaneh Feleke, Ermias Sisay Chanie, Abebe Dires Nega, Sisay Gedamu Addis, Tadila Dires Nega, Sintayehu Asnakew, Sheganew Fetene Tassaw

**Affiliations:** a Department of Pediatrics and Child Health Nursing, College of Health Sciences Debre Tabor University, P.O.BOX 272, Debre Tabor, Ethiopia; b Department of Comprehensive Nursing, School of Nursing and Midwifery, College of Medicine and Health Sciences, Wollo University, P.O. Box 1145, Dessie, Ethiopia; c Department of Comprehensive Nursing, College of Medicine & Health Sciences, Debre Tabor University, Debra tabor, Ethiopia; d Department of Psychiatry, College of Health Sciences Debre Tabor University, P.O.BOX 272, Debre Tabor, Ethiopia

**Keywords:** Chronic illnesses, Northeast Ethiopia, Patients, Risk Perception, COVID-19

## Abstract

**Introduction:**

Sever Acute Respiratory Syndrome Corona Virus 2 infections have been sharply rising in Ethiopia, and the virus significantly increases morbidity and fatality rates in patients with coexisting chronic conditions. Therefore, the purpose of this study was to evaluate Risk perception of patients with chronic illnesses toward the SARS-CoV-2 in northeastern Ethiopia in 2020.

**Methods:**

From July 21 to August 5, 2020, a cross-sectional study was carried out in a hospital setting with individuals who had chronic illnesses. The study populations included all patients with chronic conditions. The study employed multivariable logistic regression analysis to determine the factors that influence patients' evaluation of the Sever Acute Respiratory Syndrome Corona Virus 2 as low-risk.

**Results:**

Four hundred thirteen chronic illness patients participated in this study. Overall, individuals' perceptions of their risk of contracting the Sever Acute Respiratory Syndrome Corona Virus 2 were low in 37.3 % (95 % CI: 32.4–41.9). The results of multivariable analysis showed that low perception of risk towards the outcome was strongly correlated with young adults (AOR = 2.21; 95 % CI: 1.26–3.87), female sex (AOR = 2.16; 95 % CI: 1.37–3.42), and face mask nonusers (AOR = 2.17; 95 % CI: 1.35–3.49).

**Conclusion:**

According to this survey, a significant number of patients thought the pandemic posed little risk. The sense of low-risk towards the disease was independently predicted by being female, being a young adult, and not wearing face masks. Patients with chronic illnesses must get focused and enhanced health education in order to lower their elevated risk of morbidity and death.

## Highlights

• Individuals' perceptions of their risk of contracting the SARS-CoV-2 were low in 37.3%
• From the results a significant number of patients thought the pandemic posed little risk.
• From the total participants, 8.2% were extremely concerned about SARS-CoV-2.
• In the study, almost 36% of participants said they would probably get the illness.

## 1 Introduction

SARS-CoV-2 is the cause of Corona Virus Disease 2019 (COVID-19), which was officially classified as a pandemic on March 11, 2020 (Coronavirus disease, 2019). As of May 14, 2021, the World Health Organization (WHO) had received reports of over 160 million confirmed cases and 3.3 million deaths worldwide (World Helath Organization. WHO Coronavirus (COVID-19) Dashboard: retrived at 14 May, 2021).

Ethiopia announced the first coronavirus case that was confirmed on March 13, 2020. (Fmoh, 2020). Consequently, from March to April of 2020, the number of COVID-19 cases climbed gradually (Baye, 2020). However, there was a 56 % increase in new SARS-CoV-2 infections and a 12 % increase in mortality between August 10 and August 16, 2020 (WHO, 2020). Four thousand deaths and more than 264,000 confirmed cases had been reported as of May 14, 2021 (World Helath Organization. WHO Coronavirus (COVID-19) Dashboard: retrived at 14 May, 2021).

The importance of comprehending public perception of risk is growing as the number of disease-related deaths rises globally. Governmental measures now in place range from social separation and hygienic guidance to total community lockdowns. These steps are intended to keep the nation's health systems from being overburdened by an unexpected spike in cases. Perception of risk of patients includes degree of pandemic severity, public health communications, availability of healthcare, sociocultural elements, and faith in medical systems. COVID-19 is a contagious illness that spreads to vulnerable people via aerosols and respiratory droplets (Al-Qahtani, 2020). Not every SARS-CoV-2 infected person will exhibit the disease's clinical symptoms (Getaneh, 2020). Most instances start out with minor symptoms similar to the flu (Rasmussen, 2020).

Acute respiratory distress syndrome, organ failure, and death are among the severe symptoms of the disease that only a small percentage of SARS-CoV-2 infected individuals may experience (De Felice, 2020); (Ortiz-Prado, 2020) . Diabetes, chronic lung diseases, and cardiovascular diseases are the primary medical conditions linked to COVID-19-related illness and death (Stokes, 2020). By 2019, there were about 20.7 million HIV-positive individuals living in Southern and Eastern Africa (Global, 2019). Over the past few decades, there has been an increase in the prevalence of chronic diseases, especially cardiovascular disorders, in Ethiopia (Girum, 2020). HIV infections and tuberculosis are two other prevalent health issues in Ethiopia (Annabel, 2019). In spite of alternative preventative measures, a number of vaccinations have been created and are being used globally to stop SARS-CoV-2 (World Helath Organization. Coronavirus disease (COVID-19): Vaccines:(retrived at May 17, ,2021). However, the primary approaches that have been employed in numerous contexts are symptomatic and supportive therapies (World Health Organization, 2020). The pandemic has had a significant impact on the political, social, and economic challenges of nations ever since it first began to spread (Kanu, 2020). It also had a significant impact on Ethiopia's and other countries' health systems in Africa (Davies, 2020). It was difficult for developing nations to give critically ill individuals the treatment and support they needed (Basnet, 2011). Therefore, in countries with limited resources, primary prevention techniques are the most viable and optimal choices (Ali and Alharbi, 2020). The primary advised behaviors to prevent SARS-CoV-2 were staying at home, keeping a physical distance, washing hands, using face masks, and consulting a doctor (World Health Organization. Rational use of personal protective equipment for coronavirus disease (COVID-19): interim guidance (27 February, 2020).

According to community-based research carried out in Ethiopia, most urban residents were well-informed about COVID-19 and its preventative measures (Bekele, 2021). Despite the rise in instances, community adherence to COVID-19 preventative techniques and behaviors was found to be inadequate (Mola, et al., 2020).

The low participation and usage of COVID-19 preventive measures was mostly predicted by people's negative perceptions of the virus (Wise, 2020). In Ethiopia, the number of people infected with SARS-CoV-2 has been sharply raising even with disease mitigation measures in place (Baye, 2020). Limited studies have been conducted at institution level regarding to risk perception of patients with chronic illnesses toward the SARS-CoV-2 in northeastern in Ethiopia; particularly in the study area. Therefore, this study was assessed risk perception of patients with chronic illnesses toward the SARS-CoV-2 in northeastern in Ethiopia.

## 2 Methods

### 2.1 Study design and period

From July 21 to August 5, 2020, a cross-sectional study was carried out in a hospital setting with individuals who had chronic illnesses.

### 2.2 Study area

Patients with chronic illnesses who visited particular hospitals in Dessie town, in the South Wollo zone, Northeast Ethiopia, participated in the study. There are 151,094 people living in the town, 78,203 of them are women (EFDR, Population census commision. Summary and Statistical Report of the, 2007). There are three private and two public hospitals in the town that serve the people of Dessie town and the surrounding areas. Additionally, it offers medical assistance to individuals traveling from the Afar and Tigray regions.

### 2.3 Sample size and sampling procedure

The single population proportion method was used to estimate the sample size for this study, taking into account the following assumptions: a 50 % proportion, a 95 % confidence level (Z = 1.96), a 5 % margin of error, and a 10 % non-response rate, then after 10 % non-response adjustment, 384/0.9 = 427 clients in all were scheduled to participate in the study.

$$ni=\frac{\left(Z\;\alpha /2)2\;p\;(1-P\right)}{d^{2}}=\frac{\left(1.96\right)2*\;0.5\left(1-0.5\right)\;}{0.05^{2}}=\;384$$

Using a straightforward random sample approach, three of the town's five hospitals—Dessie referral hospital, Ethio, and Selam general hospitals—were chosen. Following that, an estimation of the daily patient volume in the inpatient and outpatient departments of the chosen institutions was performed, and a proportionate allocation was determined. Consequently, plans were made to investigate 295, 67, and 60 patients from Dessie Referral Hospital, Ethiopia, and Selam General Hospitals, respectively. Finally, study subjects were chosen using a successive sampling procedure.

### 2.4 Study populations

The study populations included all patients with chronic conditions such as hypertension, diabetes mellitus, chronic heart disease, chronic kidney disease, HIV/AIDS, asthma, chronic obstructive pulmonary disease, tuberculosis, cancer, and nerve diseases who were either inpatients or outpatients at specific hospitals during the data collection period. Patients in critical condition and those under the age of eighteen were not allowed to participate in the trial.

### 2.5 Data collection tool and measurement

The data was gathered using a standardized questionnaire that was pre-tested and given by an interviewer. After examining several literatures, the data collection technique was modified to include sociodemographic traits, clinical profiles of patients, risk assessments, and health-seeking behaviors of patients regarding SARS-CoV-2 (Bekele, 2021).

Six items make up the last component of the questionnaire, which was used to gauge how individuals with chronic conditions perceived their risk of contracting SARS-CoV-2. It was evaluated using a validated instrument that was modified from an earlier study and had a pooled Cronbach Alpha of 0.72 across nations (Dryhursta, 2020).

The risk perception tool for SARS-CoV-2 included questions regarding how dangerous the disease is perceived to be, how likely it is to be contracted, how concerned you are about the coronavirus, and how likely it is that friends and relatives will get sick. Those six things were: 1. How concerned are you right now about the coronavirus/COVID-19? 2. In the next six months, how probable do you believe it is that you will be directly and personally impacted by the coronavirus/COVID-19? 3. In the next six months, how likely do you believe it is that your friends and family in Ethiopia will be directly impacted by the coronavirus? 4. How much do you agree or disagree with the statement that “not many Ethiopians will be affected by the coronavirus/COVID-19″? 5. Regarding the statement, ”you will probably get sick with the coronavirus/COVID-19,“ how much do you agree or disagree? 6. To what extent do you agree or disagree that ”COVID-19 infection can be serious“?

Item one, two and three had seven Likert scale responses that ranges from “not at all” (code = 1) to “very likely”/ “very worried” (code = 7). While, item four, five and six have a five Likert scale response that ranges from “strongly disagree” (code = 1) to “strongly agree” (code = 5). For the purpose of analyses, those who responded: 1–3 = were categorized as “not worried/ not likely’’, 4= “undecided” and 5–7= “likely/worried”. For each question; those who scored “not worried/not likely’’ and “undecided” were classified to have low risk perception and those who scored “5–7” were considered to have high risk perception towards SARS-CoV-2. However, those who responded “strongly disagree”, “disagree” and “neutral” were considered as having low risk perception and those who responded “agree” and “strongly agree” were considered as having high risk perception to each question.

### 2.6 Data quality control

Following are the steps that have been followed to ensure the data quality. Supervisors and data collectors received training on COVID-19 methods to prevent infection throughout the data collecting period, as well as the study's goal and ethical considerations.

### 2.7 Operational definitions

**High risk perception of SARS-CoV-2 infection**: Patients were classified as having a high-risk perception of the pandemic if they gave the response “likely or agreed” to four or more risk perception assessment questions.

**Low risk perception of SARS-CoV-2 infection**: Patients were classified as having low risk perception towards the disease if they gave a “likely or agreed” response to three or fewer risk perception assessment questions, and as “not likely, disagree, or undecided” response to four or more perception assessment questions.

### 2.8 Data analysis

For additional statistical analysis, data was imported from EpiData version 4.6.0.0.96 and exported to IBM SPSS statistics version 25. Continuous variables were summarized using mean and standard deviation, while data were summarized using frequency, percentage, and tables. Initially, the degree of correlation between independent variables and the perceived level of danger from SARS-CoV-2 was assessed using binary logistic regression analysis. In the multivariable logistic regression analysis model, factors with a p-value of less than 0.25 from the binary logistic analysis were adjusted. At a p-value of less than 0.05, variables related to low-risk perception of SARS-CoV-2 were found to be strongly correlated in multivariable analysis.

## 3 Results

### 3.1 Socio-demographic, risk assessment and clinical characteristics of study participants

Four hundred thirteen chronic illness patients participated in this study; their mean age was 48.2 years (Standard deviation ± 15.8 years). Of the participants, over two thirds (64.9 %) lived in cities, and 22.8 % had hypertension. Regarding the current pandemic's risk assessment, no one had ever had contact with a case of SARS-CoV-2 infection. Nonetheless, in the previous two weeks, respiratory problems were reported by 9.9 % of patients.

### 3.2 Risk perception of patients towards SARS-CoV-2

In this study, Of the participants, 8.2 % were extremely concerned about SARS-CoV-2, whereas 13 were not concerned about the disease at all. 6.1 % of research participants believed they would most likely be personally impacted by the illness, while 4.6 % of patients believed COVID-19 would most likely have an impact on their friends and family. Out of all the patients, 181 (43.8 %) thought it unlikely that they would be personally and directly impacted by SARS-CoV-2 in the next six months. However, almost 36 % of participants said they would probably get the illness.

A little over 59 % of participants felt that SARS-CoV-2 will not have a significant impact on the Ethiopian population. In terms of disease morbidity, 60 % of patients thought they were more likely to have SARS-CoV-2 than 80 participants (19.4 %) thought they were unlikely to become ill with the virus. Furthermore, roughly 185 individuals (44.8 %) believed that contracting SARS-CoV-2 can be a dangerous illness. About 18.2 % of patients, however, disagreed about how serious the illness was. Overall, 154 participants (37.3 %; 95 % CI: 32.4–41.9) believed there was little risk associated with SARS-CoV-2.

### 3.3 Factors associated with Low-Risk perception of patients towards SARS-CoV-2

Using multivariable logistic regression analysis, patients' perceptions of SARS-CoV-2 as low-risk were substantially correlated with young adults' gender and lack of use of face masks (Table 1c).

**Table 1:** Factors associated with low-risk perception of patients towards the severe acute respiratory syndrome coronavirus 2 in Northeast Ethiopia, 2020 (n = 413).

**Characteristics**	**Risk perception**		**COR (95 % CI)**	**AOR (95 % CI)**

	**towards SARS-CoV-2**			
	**Low**	**High**		
**Age (in years)**				
18–34	68(48.9)	71(51.1)	1.95(1.21-	2.21(1.26-
35–54	38(29.7)	90(70.3)	3.15) *	3.87) *
>=55	48(32.9)	98(67.1)	0.86(0.51–1.44)	1.10(0.62–1.96)
			1	1
**Marital status**				
Single	33(38.4)	53(61.6)	1.10(0.43–2.79)	
Married	103(35.9)	184(64.1)	0.99(0.42–2.33)	
Divorced	9(60.0)	6(40.0)	2.66(0.71–9.95)	
Widowed	9(36.0)	16(64.0)	1	
**Sex**				
Male	56(28.3)	142(71.7)	1	1
Female	98(45.6)	117(54.4)	2.12(1.41-	2.16(1.37-
			3.12) *	3.42) *
**Residence**				
Urban	90(33.6)	178(66.4)	1	1
Rural	64(44.1)	81(55.9)	1.56(1.03–2.36) *	1.26(0.78–2.05)
**Educational status**				
No formal education	77(40.3)	114(59.7)	2.29(1.07-	1.99(0.84–4.71)
Primary school	37(40.7)	54(59.3)	4.92) *	2.32(0.94–5.72)
Secondary school	30(34.5)	57(65.5)	2.33(1.02-	1.36(0.54–3.37)
College and above	10(22.7)	34(77.3)	5.28) *	1
			1.78(0.78–4.11)	
			1	
**Family size (in number)**				
01-Mar	72(43.1)	95(56.9)	1.51(1.01-	1.38(0.87–2.21)
≥4	82(33.3)	164(66.7)	2.27) *	1
			1	
**Occupation**				
Housewives	23(37.7)	38(62.3)	1.43(0.54–3.81)	
Employed	28(32.9)	57(67.1)	1.16(0.45–2.99)	
Students	60(37.7)	99(62.3	1.43(0.59–3.49)	
Farmer	11(52.4)	10(47.6)	2.61(0.79–8.58)	
Unemployed	24(40.0)	36(60.0)	1.58(0.59–4.19)	
Merchant	8(29.6)	19(70.4)	1	
**Duration of chronic disease (in years)**				
<5	128(41.7)	179(58.3)	2.62(1.21-	1.88(0.79–4.45)
05-Oct	17(26.6)	47(73.4)	5.67) *	0.92(0.33–2.57)
>10	9(21.4)	33(78.6)	1.32(0.52–3.33)	1
			1	
**Presence of additional illness?**				
Yes				
	11(17.5)	52(82.5)	1	1
No	143(40.9)	207(59.1)	3.26(1.64–6.47) *	2.14(0.98–4.67)
**Presence of respiratory symptoms in the last two weeks?**				
Yes				
No	7(17.1)	34(82.9)	1	1
	147(39.5)	225(60.5)	3.17(1.37–7.34) *	2.02(0.77–5.23)
**Travel history in the last 2 weeks?**				
Yes				
			
No	3(18.8)	13(81.2)	1	
			
	151(38.0)	246(62.0)	2.66(0.74–9.48)	
**Wear face mask?**				
Yes	75(28.7)	186(71.3)	1	1
	79(52)	73(48)	2.68(1.77–4.06) *	2.06(1.30–3.27) *
No				
**Number of house room (in numbers)**				
1	31(37.3)	52(62.7)	1	
			
2	42(42.9)	56(57.1)	1.25(0.69–2.28)	
			
≥3	81(34.9)	151(65.1)	0.90(0.53–1.51)	
**Member of community health insurance**				
Yes				
			
No	73(37.6)	121(62.4)	1.02(0.68–1.53)	
			
	81(37.0)	138(63.0)	1	
**Use hand sanitizers?**				
				
Yes	29(26.4)	81(73.6)	1	1
				
No	125(41.3)	178(58.7)	1.96(1.21–3.17) *	1.60(0.91–2.79)
**Had social support?**				
Yes				
	129(38.9)	202(61.1)	1.42(0.84–2.39)	
			
No	25(30.9)	56(69.1)	1	

*Significantly associated at p-value of < 0.05 ^1^reference category COR: Crude odds ration AOR: Adjusted odds ratio.

Patients between the ages of 18 and 34 were 2.2 times (AOR = 2.21; 95 % CI: 1.26–3.87) more likely than patients over the age of 55 to perceive SARS-CoV-2 as low-risk. In comparison to male patients, female patients had almost double the odds (AOR = 2.16; 95 % CI: 1.37–3.42) of having low risk perception toward SARS-CoV-2. In addition, compared to face mask users, nonusers were nearly twice as likely (AOR = 2.17; 95 % CI: 1.35–3.49) to perceive the pandemic as low-risk (Table 1c).

## 4 Discussion

In Ethiopia, the number of SARS-CoV-2 cases has been steadily rising every day in spite of efforts to reduce the disease. It significantly increases morbidity and death, especially in the elderly and in patients with long-term conditions (Al-Qahtani, 2020, De Felice, 2020). The primary variables influencing the application of advised illness prevention strategies were knowledge and risk perception about SARS-CoV-2 (Wise, 2020, Yıldırım, 2020), and (Iorfa, 2020). About 37.3 % of participants in this study believed that SARS-CoV-2 posed little risk. It was nearly similar to studies conducted in Nigeria (Oyetunji, 2021), Ghana (Serwaa, 2020) and India (Jose, 2020). It was, nevertheless, less than a study carried out with university students at Debre Berhan (Tadese, 2021), in a research carried out in Northwest Ethiopia that was based on community (Kabito, 2020) and in Southwest Ethiopia among waiters (Asefa, 2020). Moreover, it was lower than other Bangladeshi studies that were carried out (Wadood, et al., 2020), among Parkinson's disease sufferers in India (Prasad, 2020) and in China (Ding, 2020). There may be differences in the research populations, the data collection methods used, and the study periods between the studies conducted in Ethiopia and the other nations. In this study, individuals between the ages of 18 and 34 had 2.2 times (AOR = 2.21; 95 % CI: 1.26–3.87) higher odds of perceiving low danger when it came to SARS-CoV-2 than patients over the age of 55. Studies carried undertaken in Ethiopia's northwest and southwest using community-based methods revealed the similar correlation (Kabito, 2020, Asefa, 2020). Furthermore, this result was consistent with earlier research done in Nigeria (Iorfa, 2020), Belgium (De Coninck, 2020) and Pakistan [52). Nonetheless, research from China and Bangladesh revealed that young adults were linked to a high-risk perception of SARS-CoV-2 (Wadood, et al., 2020). Furthermore, a study among university students in Ethiopia found that young adults had a high-risk perception (Tadese, 2021). Compared to male patients, female patients had nearly twice the odds (AOR = 2.16; 95 % CI: 1.37–3.42) of having a low-risk perception about the pandemic. This result was consistent with research from Bangladesh (Wadood, et al., 2020) and Iran (Jahangiry, 0000, Rana, 2021). However, studies done in Belgium (De Coninck, 2020); Pakistan (Rana, 2021) and Turkey (Yıldırım, 2020) showed that, compared to men, women were more likely to perceive COVID-19 as carrying a significant risk. This variance may arise from variations in the socio-demographic attributes of the research subjects and the duration of the investigation. In contrast to facemask users, those who did not wear face masks were nearly twice as likely (AOR = 2.17; 95 % CI: 1.35–3.49) to believe that SARS-CoV-2 poses little risk. Our study found that 63.2 % of participants wore face masks to prevent the disease, which is a lower percentage than a study published in Pakistan (Rizwan, et al., 2020). This disparity may result from variations in the sociodemographic traits of research participants as well as variations in the accessibility of resources. People who are at a higher risk of contracting SARS-CoV-2, such as those with chronic conditions, should be concerned about the disease in general. Therefore, it is essential to regularly apply illness preventive and control measures. Patients with long-term medical conditions perceived COVID-19 as more dangerous than people without such conditions (Tadese, 2021).

## 5 Conclusion

A sizable fraction of the chronic illness patients in our study believed that SARS-CoV-2 posed little risk. Patients' low-risk perception of the disease was highly correlated with young individuals, female gender, and face mask non-users. Patients with chronic illnesses must get focused and enhanced health education in order to lower their elevated risk of morbidity and death. Furthermore, health facilities and other government agencies ought to create, disseminate, and display pamphlets that illustrate the increased susceptibility of chronically ill people to SARS-CoV-2 infection.

## 6 Ethical approval and consent to participate

The Wollo University ethics review committee granted ethical approval (Protocol No: WU/324/T-01/2020). Formal letters were used to communicate with the administration of a few chosen hospitals. Participants in the study were told that participation was completely voluntary and that they could withdraw at any moment if the questionnaire didn't feel right. Each participant provided verbal informed consent, which was accepted by Wollo University's ethical review committee and confirmed that the study was carried out in compliance with the Declaration of Helsinki. Every piece of information that was gathered was kept private.

## 7 Consent to publication

Not applicable.

## Funding

No funding organization from the public, private, or nonprofit sectors awarded a grant for this study.

## CRediT authorship contribution statement

**Dejen Getaneh Feleke:** Formal analysis, Data curation, Conceptualization. **Ermias Sisay Chanie:** Project administration, Methodology, Investigation. **Abebe Dires Nega:** Writing – review & editing, Writing – original draft, Visualization, Validation. **Sisay Gedamu Addis:** Validation, Supervision, Software, Resources. **Tadila Dires Nega:** Resources, Project administration, Methodology. **Sintayehu Asnakew:** Validation, Supervision, Software. **Sheganew Fetene Tassaw:** Writing – review & editing, Writing – original draft, Visualization, Validation.

## Declaration of competing interest

The authors declare that they have no known competing financial interests or personal relationships that could have appeared to influence the work reported in this paper.

## Data Availability

Information will be provided upon request from the relevant author.
